# Efficacy of the Protocol for Trauma Team Activation in Taipei: A Retrospective Study

**DOI:** 10.1155/emmi/9170229

**Published:** 2025-01-10

**Authors:** Wan-Lin Chen, Ju-Chi Ou, Shih-Yu Ko, Wen-Ching Li, Hon-Ping Ma

**Affiliations:** ^1^Graduate Institute of Injury Prevention and Control, Taipei Medical University, Taipei, Taiwan; ^2^Department of Neurosurgery, Shuang-Ho Hospital-Taipei Medical University, Taipei, Taiwan; ^3^TMU Neuroscience Research Center, Taipei Medical University, Taipei, Taiwan; ^4^Department of Surgery, School of Medicine, College of Medicine, Taipei Medical University, Taipei, Taiwan; ^5^Department of Emergency Medicine, Shuang-Ho Hospital-Taipei Medical University, New Taipei City, Taiwan; ^6^Department of Emergency Medicine, School of Medicine, Taipei Medical University, Taipei, Taiwan

## Abstract

**Introduction:** Trauma triage is the use of trauma assessment for prioritizing patients for treatment or transport by injury severity. According to Taiwan Public Health Report, accidents and their adverse events were the sixth leading cause of death and accounted for over 7000 casualties in 2009. However, a lack of accuracy in identifying the severity of a patient's injury and their prehospital information can result in inappropriate triage. This study evaluated the efficacy of field triage guidelines governing trauma team activation in Taipei and explored the characteristics of undertriaged and overtriaged patients.

**Methods:** This study retrospectively observed all patients with trauma transported to the emergency department of a medical center by Taipei City public ambulance from January 1, 2016, to December 31, 2019. A total of 2217 patients were included. The Cribari matrix method was used to assess undertriage and overtriage. A logistic regression was employed to analyze the effect of risk factors in patients with major trauma.

**Results:** In this study, 320 and 1897 patients with trauma had full and limited trauma team activation, respectively. Among them, 664 patients with trauma were older than 65 years, and most of them were injured in a traffic accident. Among patients with major trauma, 24, 214, and 156 patients were aged < 20, 20–65, and > 65 years, respectively. A logistic regression analysis revealed that patients with a Glasgow Coma Scale score of less than 13, with systolic blood pressure level of less than 90, and with respiratory rate over 30 breaths per minute was more likely to be appropriately triaged.

**Conclusion:** The Taipei prehospital field triage guidelines are acceptable but not an ideal tool for identifying patients with major trauma, with an overtriage rate of 48.12% and an undertriage rate of 12.03%. To decrease undertriage or overtriage rates, emergency medical service providers should receive comprehensive training.

## 1. Introduction

Trauma triage [1] involves the systematic assessment of trauma patients to prioritize them for treatment or transport based on the severity of their injuries. This process can be categorized into two primary types: field triage and hospital triage. Inaccuracies in determining the severity of a patient's injuries, as well as deficiencies in prehospital information, can lead to inappropriate triage decisions, with subsequent implications for resource utilization. Historically, two-tier activation systems for trauma teams have been implemented since the inception of trauma care systems. Initial evaluations demonstrated both the cost-effectiveness and the efficient allocation of resources associated with these systems. Consequently, there has been a notable increase in research focused on two-tier trauma team activation (TTA) models [2, 3]. Key indicators of the quality of trauma systems include rates of undertriage and overtriage [4]. Specifically, undertriage can adversely affect patient outcomes, while overtriage may divert essential resources away from other patients in need. The American College of Surgeons Committee on Trauma (ACS-COT) considers an undertriage rate of less than 5% and an overtriage rate of less than 35% to be acceptable thresholds [1]. Injury triage systems were developed to ensure that resources are judiciously allocated to trauma patients based on their clinical needs.

According to Taiwan Public Health Report, accidents and their adverse events were the sixth leading cause of death and accounted for over 7000 casualties in 2009 [5]. Taipei City Government held seven field triage training sessions for paramedics from 2015 to 2016, and field trauma training was found to improve the accuracy of field triage scheme [6]. In another study on Taipei City, no significant association was found between in-hospital mortality and TTA. However, the group subject to TTA guidelines had a higher risk of intensive care unit (ICU) admission, prolonged length of hospital stay (LOS), and prolonged ICU LOS. In addition, a subgroup analysis revealed that in the TTA group, patients aged 60–80 years, with major injury (injury severity score [ISS] ≥ 16), with clear consciousness, and with nonhead injury had a higher risk of mortality. These findings indicate that patients selected using the TTA criteria had higher ISS and a higher risk of mortality [7]. According to our review of the literature, studies have yet to evaluate the effectiveness of the field triage system. Therefore, this study evaluated the association between the Taipei Field triage Guidelines and triage appropriateness. It determined by the modified Cribari Matrix in order to determine if the Taipei Field Triage Guidelines may need to be modified to more appropriately utilize hospital resources.

## 2. Methods

### 2.1. Data

We explored the accuracy of Taipei prehospital field triage guidelines in identifying patients with major trauma (using ISS ≥ 16) and potential factors that may affect accuracy.

Taipei prehospital field triage guidelines comprise 4 major criteria with 11 items each. The four major criteria are vital signs, anatomic injuries, injury mechanism, and special considerations. If a patient with on-site trauma meets more than one criterion, the patient must be sent to a first-aid hospital, and the on-site team must notify the hospital to activate the trauma team. Patients with major trauma were classified as patients having an ISS of ≥ 16. We hypothesized that Taipei prehospital field triage guidelines could be used to accurately identify patients with major trauma (ISS ≥ 16).

**Figure 1:**
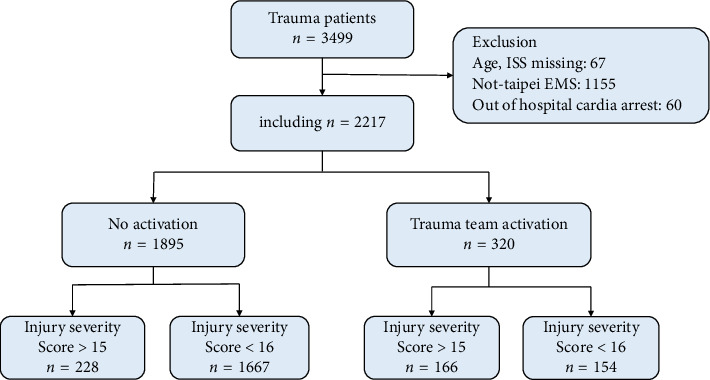
Study flowchart.

This study retrospectively observed all patients with trauma who were transported to the emergency department of a medical center by Taipei City public ambulance from January 1, 2016 to December 31, 2019. The following patients were excluded: patients who were transported by means other than a Taipei City public ambulance and patients with out-of-hospital cardiac arrest. A total of 1322 people were included in the analysis. Data were extracted from a trauma registry of the study hospital. GCS was calculated by the emergency medical technicians (EMTs) and were recorded in the medical records accordingly. ISS was calculated by nurses in the ED and recorded after thorough revaluation. For patients who died in route to the hospital or died in the ED were both still included if there was no missing data. The flowchart of patient selection is shown in Figure 1.

The Cribari matrix method (CMM) was used to assess undertriage and overtriage. We used a modified CMM for greater accuracy [8]. In our study, there was either TTA or no TTA. No partial TTA since it was ambiguous and nonbillable.

Ethical approval: This study was approved by the Institutional Review Board of Taipei Medical University (TMU-JIRB #N202107116).

### 2.2. Taipei Field Triage System

**Figure 2:**
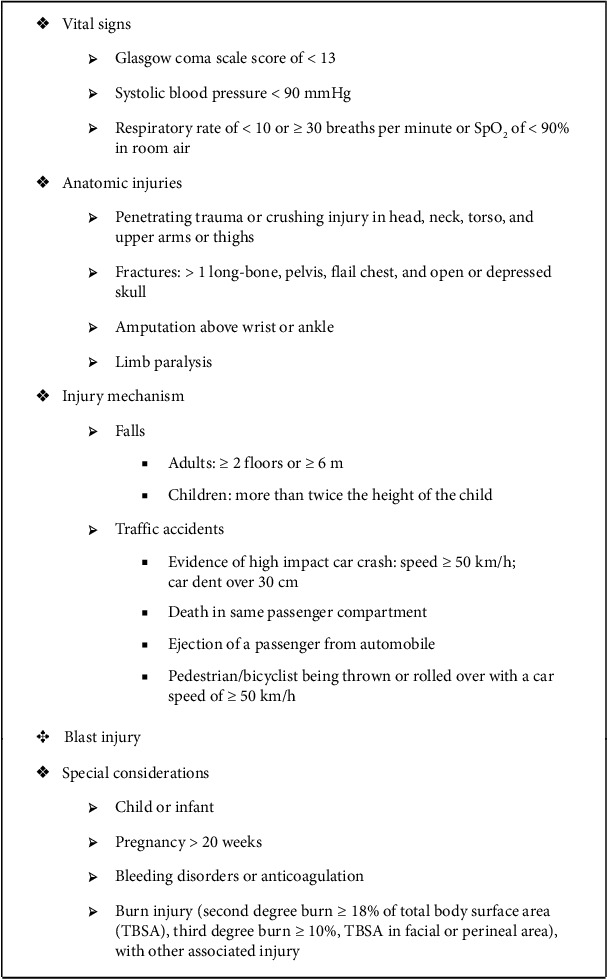
Decision scheme of Taipei TTA criteria [9].

As shown in Figure 2, Taipei prehospital field triage guidelines cover five criteria: vital signs, anatomic injuries, injury mechanism, blast injury, and special considerations. The criterion of vital signs comprise the items of having a Glasgow Coma Scale (GSC) score of < 13, systolic blood pressure (SBP) level of < 90, respiratory rate of < 10 or ≥ 30 breaths per minute, and a SpO_2_ of < 90%. The criterion of anatomic injuries comprise the items of having penetrating trauma; crushing injury in the head, neck, torso, or upper arms or thighs; fractures; amputation above the wrist or ankle; and limb paralysis. The criterion of injury mechanism comprises the items of having had a fall and having been involved in a traffic accident. The criterion of special considerations comprises the items of being a child or an infant, being pregnant, having bleeding disorders or anticoagulation, and having burn injury.

### 2.3. CMM

The first edition of the criteria of field triage was developed according to the recommendations of the ACS-COT in 1979 [1]. The current Field Triage Decision Scheme was developed according to the evidence-based review of the ACS-COT and the National Highway Traffic Safety Administration and Center for Disease Control and Prevention [9]. The original and modified version of CMM is shown in Table 1.

### 2.4. Statistical Analysis

Continuous and categorical variables are presented as median (IQR) and frequency and percentage, respectively. A logistic regression was performed to analyze the impact of risk factors in patients with major trauma. The patients' values with missing were excluded. All statistical analyses were conducted using R software. Significance was indicated by *p* < 0.05. For consistency throughout study period and to avoid confusion, patients with missing data were excluded.

## 3. Results

**Figure 3:**
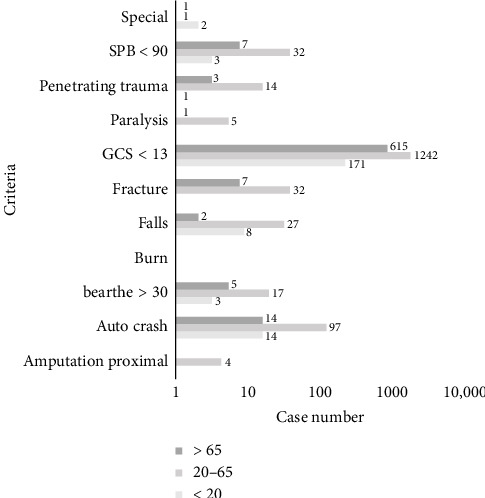
Number of patients who met TTA criteria.

Between 2016 and 2019, among patients with trauma, 195, 1,358, and 664 patients were aged < 20 years (young group), aged 20–65 years (adult group), and aged > 65 (older adult group), respectively; these three age groups significantly differed with respect to patient characteristics (i.e., sex, age, and mechanism of injury). The young and adult groups had more women than men, and the older adult group had more men than women. The average ages of young, adult and older adult groups were 15.52 (±5.03), 40.51 (±14.66), and 78.26 (±8.28) years, respectively. Over 65% of the young and adult groups' major traumas were caused by traffic accidents, whereas more than 58% of the older patients' major traumas were caused by falls. The basic and clinical characteristics of the patients are shown in Table 2. The number of patients who met the Taipei TTA criteria is shown in Figure 3.

Among the three age groups, TTA rate, average ISS, ISS higher than—16 rate, and undertriage rate significantly differed. The TTA rates of the young group and older adult group were the highest and lowest, respectively. The average ISSs of the older adult group and young group were the highest and lowest, respectively. The number of patients with an ISS of ≥ 16 in the older adult group was the highest (23.49%), and that in the young group was the lowest (12.31%). The undertriage rate of the older adult group was the highest (19%), and that of the young group was the lowest (4%).

### 3.1. Undertriage

The risk factors, gender, mechanism of injury, GCS, SBP, SpO_2_, and respiratory rate and ISS were significantly different between appropriate and undertriage groups (Table 3). The female percentage was 65% in appropriate group and 54.82% in undertriage group. The median GCS score were 8.12 and 14.86 for appropriate and undertriage groups. The median SBP score were 112 and 128 for appropriate and undertriage groups. The median SpO_2_ score were 95.38 and 97.45 for appropriate and undertriage groups. The median breathe rate were 152 and 228 for appropriate and undertriage groups.

### 3.2. Overtriage

The risk factors, age, mechanism of injury, GCS, SBP, and respiratory rate were significantly different between appropriate and overtriage groups (Table 4). The median age was 46.70 years old in appropriate group and 41.64 years old in overtriage group.

Four criteria were significantly associated with overtriage (Table 5). Patients with a GCS score of less than 13, SBP level less than 90, and respiratory rate of over 30 breaths per minute were more likely to be appropriately triaged. By contrast, the patients with fractures were more likely to be overtriaged.

## 4. Discussion

In this study, 664 patients had trauma (30%). Because Taiwan's population is aging, older patients make up a large proportion of patients with trauma [10]. We found that 320 patients notified the hospital to activate the trauma team of the fire department, and 394 patients had a diagnosis of major trauma after a physician's evaluation, which indicates that most of the patients with major trauma were not rescued by the on-site emergency medical personnel.

Taipei City Prehospital On-Site Triage Indicator can be used to send the right patient to the right hospital at the right time to improve the patient's chance of survival and prevent permanent disability. The rapid determination of whether a patient has a major trauma is challenging and requires a detailed investigation and evaluation by the hospital. In addition, other challenges include assessment of the following: the mechanism of injury on-site, the patient's vital signs and risk of their condition deteriorating, and the severity of injury and provision of immediate medical attention. Additional studies are required to investigate other factors that may be pertinent to the patient's injury, such as their age or medical history.

Accurate prehospital on-site triage indicators are essential for effective trauma evaluation, as inappropriate triage—either undertriage or overtriage—can result in significant negative outcomes. Undertriage represents a particularly critical concern in trauma care, as it may lead to delays in treatment or the misdiagnosis of injuries, ultimately contributing to increased mortality [11, 12]. In contrast, overtriage can disrupt the comprehensive management of critically ill patients, resulting in heightened healthcare costs and contributing to overcrowding in trauma centers [13].

Despite the guidelines provided by the ACS-COT regarding on-site triage, several factors can influence undertriage rates, including the judgment of EMTs, requests from family members, and variations in the presentation of serious injuries based on age [14]. None of the existing triage guidelines worldwide adequately fulfill the criteria established by the ACS-COT, which include an overtriage rate of 35%–50% and an undertriage rate of less than 5% [15]. Consequently, undertriage continues to pose a significant challenge, even in countries with advanced healthcare systems.

In this study, the undertriage rate of older patients (aged > 65 years) was 19%, which was 10% higher than that of young and adult patients (aged 20–64 years). These findings are consistent with those previous studies. Inappropriate triage of the older patients may be due to the following factors: having low impact injury mechanism (e.g., falls), receiving medication (e.g., anticoagulants), and differences in the pathophysiology of the older patients [16, 17], suggesting that older patients tend to have more cognitive and physical impairments with preexisting comorbidities. Therefore, the inappropriate triage of older patients will remain a major problem, and this warrants additional study. After all, Taiwan is less than 4 years from becoming an aging society.

The “Taipei City Prehospital On-Site Triage Index” is acceptable but not an ideal tool for identifying major traumas in patients and thus requires improvement; it has an overtriage rate of 48.12% and an undertriage rate of 12.03%. Because this index has its limitations, a considerable number of patients with major trauma do not receive proper care. Older patients (≥ 65 years old) with trauma should receive special attention from emergency medical personnel. When the on-site patient meets one of the following criteria: having a GCS score of < 13, respiratory rate of < 10 or ≥ 30 breaths per minute (or oxygen saturation of < 90% in room air), or a fall height equivalent to the height of a 2-story building, the on-site emergency medical personnel should provide quality care for patient and notify the hospital to activate the trauma team. If the hospital receives this notification, it should activate the trauma team. The overall rescue time of patients can be reduced, because the activation of the hospital trauma team can reduce the time taken to transport the patient with major trauma from an emergency room to an operating room [18], and can reduce the mortality rate [19], resulting in more efficient treatment and better outcomes for patients with major trauma.

Cribari is not necessarily the gold standard. Each methodology has its own advantages and limitations. We used CMM because it was relatively easy to extract data from medical records for further statistical analysis in the study hospital. As a result, there was inherent risk of overtriage since activation of trauma team is dichotomous (ISS more or less than 15). Patients who fulfilled our TTA criteria received multidisciplinary treatment plans and regular reviews. Patients who did not meet the TTA criteria would only be treated with the specialty needed for the injury. In the study hospital, there was no partial TTA.

Our study was the first attempt to evaluate how we used our guidelines for Taipei City prehospital paramedic team for resource optimization. At the study period between 2016 and 2020, we noted overtriage and undertriage rates were higher compared with that in the reference we cited. This provided us a clearer goal to work on as the acceptable overtriage and undertriage rates continue to evolve with increasing challenges in resources limitations. The findings of our study did validate the necessity to revise our guidelines for continuous quality improvement. Reducing undertriage generally takes precedence over decreasing overtriage, and the Dutch Health Care Institute and ACS-COT recommend to attain maximum undertriage rates of 10% and 5%, respectively. No inclusive trauma system worldwide is currently able to adhere with these guidelines while preserving acceptable overtriage rates. The ACS-COT does suggest overtriage rates up to 35% may be acceptable but no universal agreement exists on acceptable overtriage rates [20].

This study has several limitations. This research is a retrospective study, and some data were thus unavailable. Furthermore, the study setting was a single medical center (a first-aid-responsibility hospital with a heavy patient load), and the findings may not be generalizable to moderate sized and general emergency hospitals. However, because the data collected by this medical center are currently the most complete among hospitals in Taipei, we used the data of that hospital. In addition, the on-site ambulance personnel may be using different standards; however, they are subject to continual training [6]. Finally, because the ambulance personnel before arriving at the hospital were unable to identify the potential head trauma of patients, the injury mechanism could not be accurately presented. This warrants further investigation with regard to the provision of treatment for the injury site after arriving at the hospital and before arriving at the hospital.

## 5. Conclusion

To improve the overall quality of the treatment of trauma, the emergency medical personnel should continuously receive training to improve the overall quality of care. Every minute in the hospital counts, and the horizontal communication and contact before and after hospital should thus be strengthened to reduce the treatment time of patients with major trauma. Future research should include the emergency responsibility hospitals in the entire Taipei City to ensure that more samples are obtained to explore its related indicators and continue improving the accuracy and completeness of the registration data of the Taipei City Government Trauma System. More reliable data may be provided in the future.

## Figures and Tables

**Table 1:** Original and modified version of CMM for assessing triage tool.

Standard version	ISS 0-9	ISS 10-14	ISS 15-24	ISS 25-75
Full trauma team activation	OT	OT	AT	AT
Partial trauma team activation	AT	AT	AT	UT
Trauma consultation	AT	AT	UT	UT
Trauma service not notified	AT	UT	UT	UT

**Modified version**	**ISS 0–15**		**ISS 16**–**75**	

Trauma team activation	OT		AT	
No activation	AT		UT	

**Table 2:** Basic and clinical characteristics of patients from 2016 to 2020.

Characteristic	Total	No-TTA	TTA	*p* value
Sample size	2217	1897	320	
Gender^#^				< 0.01^∗^
Female	1222 (55.12%)	1005 (52.98%)	217 (67.81%)	
Male	994 (44.84%)	891 (46.97%))	103 (31.19%)	
Age, median (IQR), years	49.62 (23.52)	50.52 (23.66)	44.27 (21.98)	< 0.01^∗^
Mechanism of injury^#^				< 0.01^∗^
Fall	597 (26.93%)	572 (30.15%)	25 (7.81%)	
Vehicle incident	1415 (63.82%)	1176 (61.99%)	239 (74.69%)	
Others	205 (9.25%)	149 (7.85%)	56 (17.50%)	
ISS, median (IQR)^&^	4 (8)	4 (8)	16 (21)	< 0.01^∗^
ISS < 16	1823 (82.23%)	1669 (87.98%)	154 (48.12%)	
ISS ≥ 16, *n* (%)	394 (17.77%)	228 (12.02%)	166 (51.88%)	

Abbreviations: ISS, injury severity score; SD, standard deviation.

^#^Chi-square test.

^∗^Significant.

^&^Wilcoxon signed rank test.

**Table 3:** Basic and clinical characteristics of patients undertriage.

Characteristic	App	Under	*p* value
Sample size	166	228	
Gender^#^			0.04^∗^
Female	109 (65.66%)	125 (54.82%)	
Male	57 (34.34%)	103 (45.18%)	
Age, mean (SD), years	46.7 (22.77)	60.94 (21.64)	
Mechanism of injury^#^			< 0.01^∗^
Fall	11 (6.63%)	78 (34.21%)	
Vehicle incident	128 (77.11%)	141 (61.84%)	
Others	27 (16.27%)	9 (3.95%)	
GCS, mean (SD)	8.12 (4.53)	14.86 (0.80)	< 0.01^∗^
SBP, median (IQR)	112 (149)	128 (50)	< 0.01^∗^
SpO_2_, mean (SD)	95.38 (4.46)	97.45 (2.15)	< 0.01^∗^
Breathe rate, 10–29/min	152 (91.57%)	228 (100%)	< 0.01^∗^
ISS, median (IQR)^&^	25 (12)	18 (5)	< 0.01^∗^

Abbreviations: GCS, Glasgow Coma Scale; ISS, injury severity score; SBP, systolic blood pressure; SD, standard deviation.

^#^Chi-square test.

^∗^Significant.

^&^Wilcoxon signed rank test.

**Table 4:** Basic and clinical characteristics of patients with major traumas with full TTA.

Characteristic	Appropriate triage *n* = 166	Overtriage *n* = 154	*p* value
Age, median (IQR), year	46.70 (22.77)	41.64 (20.85)	0.04
Age group^#^			0.09
< 20	17 (10.24%)	19 (12.34%)	
20–65	107 (64.46%)	111 (72.08%)	
> 65	42 (25.30%)	24 (15.58%)	
Male, *n* (%)^#^	57 (34.34%)	46 (29.87%)	0.46
Mechanism of injury^#^			0.55
Falls	11 (6.63%)	14 (9.09%)	
Motor vehicle/collision	128 (77.11%)	111 (72.08%)	
Others	27 (16.27%)	29 (18.83%)	
ISS, median (IQR)^&^	25 (12)	4 (7)	< 0.01^∗^

Abbreviations: ISS, injury severity score; SD. standard deviation.

^#^Chi-square test.

^∗^Significant.

^&^Wilcoxon signed rank test.

**Table 5:** Results of univariate logistic regression analysis for the 10 criteria among patients with full TTA (outcome: overtriage vs. appropriate triage).

Variable	Number overtriage (*n* = 154)	Not (*n* = 166)	OR	95% CI of OR	*p* value
GCS < 13	56	130	0.16	(0.10, 0.26)	< 0.01
SBP < 90^∗^	15 (140)	22 (137)	0.50	(0.25, 0.97)	0.04
Breathe < 30	3	22	0.15	(0.05, 0.47)	< 0.01
Head	9	9	1.08	(0.43, 2.74)	0.87
Fracture	26	13	2.34	(1.17, 4.70)	0.02
Extreme	3	1	2.55	(0.37, 17.48)	0.31
Paralysis	2	4	0.59	(0.12, 2.82)	0.47
Fall	15	22	0.71	(0.36, 1.42)	0.33
Crash	68	57	1.51	(0.96, 2.36)	0.07
Special	2	2	1.08	(0.18, 6.31)	0.94

^∗^SBP had missing values, only 140 and 137 patients in overtriage group and nonovertriage group, respectively.

## Data Availability

The data used to support the findings of this study are available from the corresponding author upon request.
